# Mapping microRNA expression quantitative trait loci in the prenatal human brain implicates miR-1908-5p expression in bipolar disorder and other brain-related traits

**DOI:** 10.1093/hmg/ddad118

**Published:** 2023-07-20

**Authors:** Carolina C Toste, Michael C O’Donovan, Nicholas J Bray

**Affiliations:** Centre for Neuropsychiatric Genetics & Genomics, Division of Psychological Medicine & Clinical Neurosciences, Cardiff University, Cardiff CF10 4HQ, United Kingdom; Centre for Neuropsychiatric Genetics & Genomics, Division of Psychological Medicine & Clinical Neurosciences, Cardiff University, Cardiff CF10 4HQ, United Kingdom; Centre for Neuropsychiatric Genetics & Genomics, Division of Psychological Medicine & Clinical Neurosciences, Cardiff University, Cardiff CF10 4HQ, United Kingdom; Neuroscience & Mental Health Innovation Institute, Cardiff University, Cardiff CF24 4HQ, United Kingdom

## Abstract

MicroRNA (miRNA) are small non-coding RNA involved in post-transcriptional gene regulation. Given their known involvement in early neurodevelopment processes, we here sought to identify common genetic variants associated with altered miRNA expression in the prenatal human brain. We performed small RNA sequencing on brain tissue from 112 genome-wide genotyped fetuses from the second trimester of gestation, identifying high-confidence (false discovery rate < 0.05) expression quantitative trait loci for 30 mature miRNA. Integrating our findings with genome-wide association study data for brain-related disorders, we implicate increased prenatal expression of miR-1908-5p as a risk mechanism for bipolar disorder and find that predicted mRNA targets of miR-1908-5p that are expressed in the fetal brain are enriched for common variant genetic association with the condition. Extending these analyses to other brain-related traits, we find that common genetic variation associated with increased miR-1908-5p expression in fetal brain is additionally associated with depressive symptoms, irritability, increased right cerebellum exterior volume and increased sleep duration in the general population. Our findings provide support to the view that altered miRNA expression can influence susceptibility to neuropsychiatric illness and suggest an early neurodevelopmental risk mechanism for bipolar disorder.

## Introduction

MicroRNA (miRNA) are small (approximately 22 nucleotide) non-coding RNAs that regulate the expression of other genes by binding to their mRNA and causing translational repression and/or mRNA destabilization (1). More than 2600 miRNAs have been identified in humans (2), the majority transcribed as primary miRNAs (pri-miRNAs) before being processed into shorter pre-miRNA in the nucleus and then cleaved into the mature miRNA after export to the cytoplasm (3). Around half of known miRNA are expressed in the mammalian brain (3), where they contribute to a variety of processes including cell proliferation, neural differentiation, neuron migration and synapse formation (4).

Effects on gene expression are recognised as a principal mechanism by which common genetic variation influences complex traits, including susceptibility to neuropsychiatric disorders (5,6). Genetic variants that are associated with altered gene expression can be mapped on a genomic scale (as expression quantitative trait loci, or eQTL) by combining genome-wide genotyping with transcriptome profiling of a tissue of interest (7). Identified eQTL can then be integrated with genome wide association study (GWAS) statistics for a given trait to identify genes that potentially influence that trait through differences in their expression (8).

miRNA have been suggested to be involved in neuropsychiatric disorders based on their location in genome-wide significant risk loci (9–11) and differential expression in patient samples (12–14). However, few studies have attempted to map genetic variants associated with miRNA expression in the human brain (11, 15–17). Of these, only one published study has been conducted using prenatal human brain tissue (17). Given that miRNA play documented roles in a variety of neurodevelopmental processes (4), and evidence for altered gene regulation in the prenatal brain in adult-onset psychiatric as well neurodevelopmental disorders (18–20), we here sought to identify miRNA eQTL in an independent collection of 112 human fetal brain samples from the second trimester of gestation. We identified high-confidence eQTL for 30 miRNA, of which 23 have not been previously reported in the developing human brain (17). We further integrated these data with large-scale GWAS data for neuropsychiatric disorders and other brain-related phenotypes, providing evidence that expression of miR-1908-5p mediates risk for bipolar disorder and other traits.

## Results

### Identification of miRNA *cis*-eQTL in the developing human brain

We performed small RNA sequencing on brain tissue from 112 unrelated fetuses from the second trimester of gestation, deriving expression measures for 1449 miRNA. After genotype imputation and strict quality control, 5.3 million SNPs were available for analysis. *cis*-eQTL analysis of miRNA expression was performed using SNPs within 500 kb either side of the first base of each mature miRNA using FastQTL (21), accounting for covariates. We thus identified *cis*-eQTL for 30 miRNA at a false discovery rate (FDR) < 0.05 (Table 1). The most significant eQTL for seven of these miRNA was either the same SNP or in linkage disequilibrium (*r*^2^ > 0.5 across populations) with the most significant eQTL for the same miRNA in a recent study (17). The remaining 23 high-confidence miRNA eQTL have not previously been reported in fetal brain.

**Table 1:** The most significant *cis-*eQTL for 30 miRNA in human fetal brain identified in this study (FDR < 0.05)

miRNA	Chr	Top miR-eQTL	Distance to mature miRNA (bp)	*q*-value
miR-5683^*^	chr6	rs7769202	79	8.91E-13
miR-4707-3p^*^^*^	chr14	rs2273626	15	3.21E-12
miR-544b	chr3	rs3821536	32 618	1.80E-08
miR-6868-3p	chr17	rs2243486	−10 072	1.80E-08
miR-618^*^	chr12	rs10862209	−2621	2.83E-08
miR-1908-5p	chr11	rs174561	26	9.27E-08
miR-4326^*^	chr20	rs7263455	15 722	5.85E-07
miR-3615^*^	chr17	rs745666	-5	6.43E-07
miR-548ba^*^	chr2	rs2140551	−464 362	7.40E-07
miR-1269a	chr4	rs72641631	−6625	2.32E-06
miR-1287-5p	chr10	rs942803	13 602	1.63E-05
miR-3117-3p	chr1	rs1925342	21 061	5.98E-05
miR-4467	chr7	rs6971245	−3908	6.95E-05
miR-4803	chr5	rs1561401	−450	2.19E-04
miR-323b-3p	chr14	rs56103835	−51	2.19E-04
miR-7854-3p	chr16	rs2927318	−303	2.43E-04
miR-4662a-5p	chr8	rs7006762	−82 859	8.58E-04
miR-548at-5p	chr17	rs11653901	−10 624	1.05E-03
miR-3161	chr11	rs74236456	−66 010	1.23E-03
miR-3125	chr2	rs6717278	6563	2.07E-03
miR-3176	chr16	rs116698525	−15 147	2.66E-03
miR-1270	chr19	rs28576121	−18 408	2.77E-03
miR-4761-3p	chr22	rs4680	−67	3.25E-03
miR-641^*^^*^	chr19	rs41275750	−50 416	0.016
miR-4423-5p	chr1	rs709777	−13 198	0.016
miR-6886-5p	chr19	rs1003723	25	0.031
miR-202-5p	chr10	rs11101657	−3583	0.039
miR-6840-5p	chr7	rs112622797	−36 864	0.039
miR-6826-3p	chr3	rs6788178	54 097	0.039
miR-3938	chr3	rs55852613	−23 443	0.041

^*^The same SNP identified as the most significant eQTL (FDR < 0.05) for the same miRNA by Lafferty et al. (17). ^*^^*^Most significant SNP in linkage disequilibrium (*r*^2^ > 0.5 across populations) with the most significant eQTL (FDR < 0.05) for the same miRNA in the study of Lafferty et al. (17).

**Figure 1:**
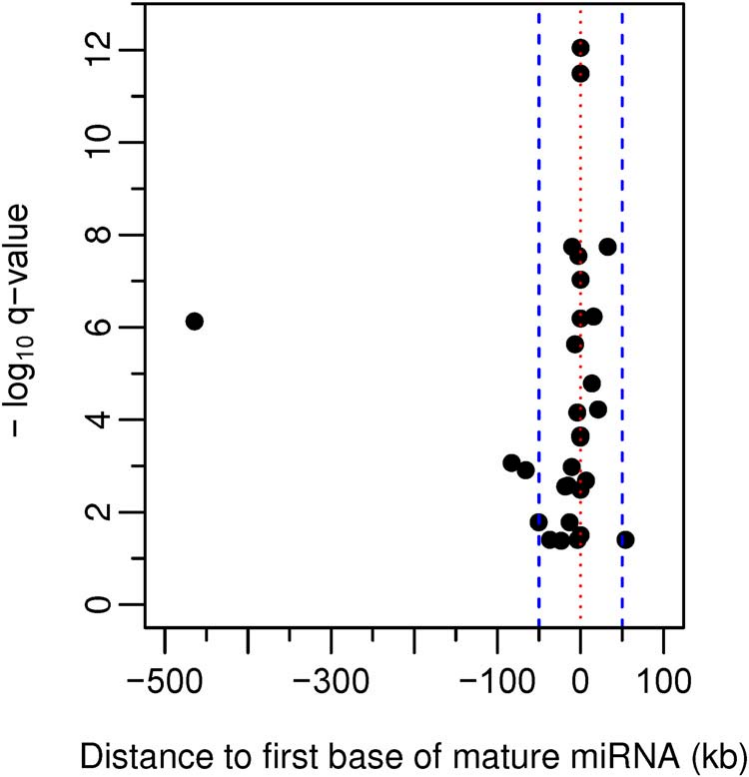
Genomic distance of top eQTL (FDR < 0.05) from sequence encoding their associated mature miRNA. The *X*-axis indicates kilobases (kb) to the first base of the sequence encoding the mature miRNA (indicated by the dotted line). The dashed lines indicate 50 kb up- and down-stream of sequence encoding the mature miRNA. The *Y*-axis indicates the −log_10_  *q*-value of the eQTL.

The most significant eQTL for 25 (83%) of the 30 variably *cis*-regulated miRNA identified in this study was within 50 kb of its mature miRNA sequence, with only one top eQTL detected more than 100 kb from its associated miRNA (Fig. 1). The most significant eQTL for 5 of these miRNA was located either within the mature miRNA sequence itself (rs2273626 in miR- 4707-3p) or in other parts of the pri-miRNA hairpin (rs745666 in miR-3615, rs1003723 in miR-6886, rs174561 in miR-1908 and rs56103835 in miR-323b). All but one of these five SNPs (associated with miR-6886-5p expression) was predicted to alter pri-miRNA hairpin secondary structure (Supplementary Material, Fig. S1). Given its position in the mature miRNA sequence, we confirmed that the association between genotype at rs2273626 and expression of miR-4707-3p (also implicated as an eQTL for miR-4707-3p in the study of Lafferty and colleagues [ref. 17] through an SNP in strong linkage disequilibrium) was not due to sequence alignment bias by repeating the analysis, allowing one mismatch in alignment to both miRbase and the human genome (FDR of rs2273626 as an eQTL for miR-4707-3p in the repeated analysis = 4.27 × 10^−12^).

Twenty-two (73%) of the miRNA identified as having high-confidence eQTL in this study were located within either introns or exons of known mRNA genes (Supplementary Material, Table S1). We tested whether identified miR-eQTL are additionally eQTL for their host genes using data from our previous mRNA-based eQTL analysis in second trimester fetal brain (18) which used largely overlapping (*N* = 76) samples. Only the eQTL for miR-4707-3p (rs2273626) was even nominally significantly associated with expression of its host gene (*HAUS4*; nominal *P* = 0.0002), indicating that most of the miR-eQTL identified in this study operate through independent mechanisms.

### Association between miRNA *cis*-eQTL and brain-related traits

**Figure 2:**
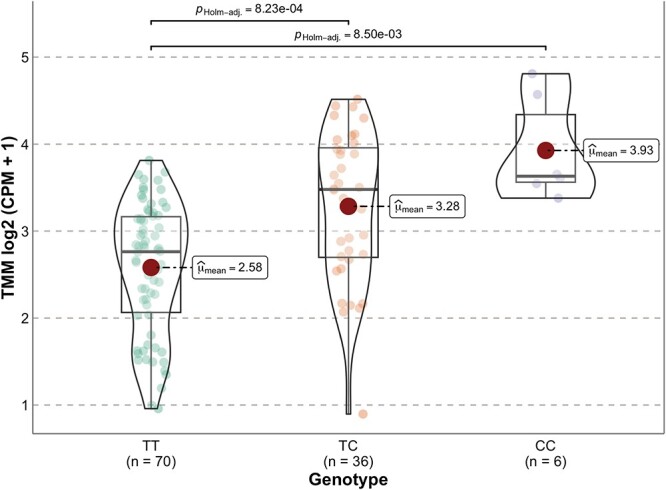
Association between genotype at SNP rs174561 and expression of miR-1908-5p in second trimester fetal brain. The thicker horizontal line in each boxplot indicates the median miR-1908-5p expression for each genotype and the large dot indicates the mean expression of miR-1908-5p for each genotype.

miR-eQTL operating in the developing human brain could potentially influence susceptibility to later developmental, neurological and psychiatric disorders. We initially screened GWAS summary data for 11 such conditions (Alzheimer’s disease [22], anorexia [23], anxiety disorders [24], attention-deficit hyperactivity disorder [25], autism [26], bipolar disorder [27], depression [28], obsessive-compulsive disorder [29], post-traumatic stress disorder [30], schizophrenia [31] and Tourette Syndrome [32]) for evidence of association with the most significant eQTL for the 30 variably *cis*-regulated miRNA identified in this study (Bonferroni-corrected *P*-value threshold for 11 traits and 30 eQTL = 1.5 × 10^−4^). We found that SNP rs174561, the most significant eQTL for miR-1908-5p (and located within its pri-miRNA hairpin), is also strongly associated with bipolar disorder (*P* = 1.83 × 10^−11^). There were also nominally significant associations between this SNP and depression (*P* = 1.35 × 10^−3^) and schizophrenia (*P* = 0.03). The risk (C-) allele for bipolar disorder (and potentially the other two conditions) is associated with increased miR-1908-5p expression in the second trimester fetal brain (Figure 2). No other miR-eQTL reached the Bonferroni threshold for association with any of the tested conditions (Supplementary Material, Table S2).

To investigate potential pleiotropic effects of miR-1908-5p eQTL rs174561 on other brain-related phenotypes, we performed a Phenome-Wide Association study (PheWas) using the Atlas of GWAS Summary Statistics (33), which includes GWAS data for more than 800 psychiatric, neurological and cognitive traits. Limiting our analyses to these domains, we found that the C-allele of rs174561 is additionally associated with depressive symptoms (34), cognitive performance (35), exterior cerebellar volume (36), intelligence (37), irritability (38) and sleep duration (39) in general population-based samples at the Bonferroni-corrected threshold for 836 tested traits (*P <* 6.0 × 10^−5^).

**Figure 3:**
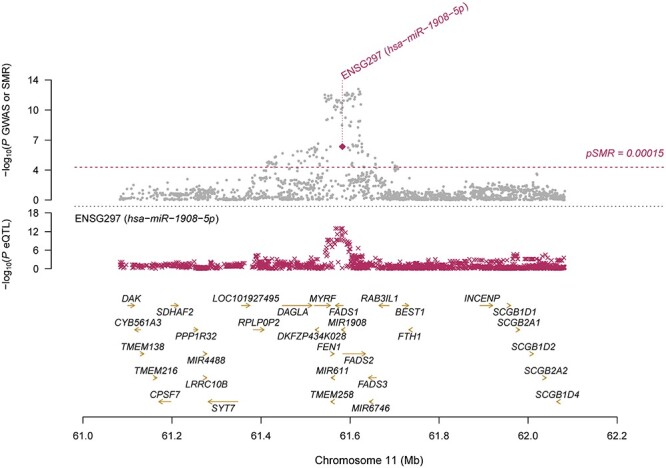
SMR analysis implicating miR-1908-5p expression in bipolar disorder. Top plot: dots represent −log_10_  *P*-values for association between SNPs at the chromosome 11 (61–62 Mb) locus and bipolar disorder in the GWAS of Mullins et al. (27). The diamond represents the −log_10_  *P*-value of miR-1908-5p expression in the SMR test and the dashed line indicates the Bonferroni-corrected *P*-value threshold for testing 30 miRNA across 11 traits. Bottom plot: crosses represent the −log_10_  *P*-values for associations between SNPs at the chromosome 11 (61–62 Mb) locus and miR-1908-5p expression in fetal brain in this study. Genomic coordinates are hg19.

To more formally test whether associations with bipolar disorder and other brain-related traits are potentially mediated by miR-1908-5p expression, we performed Summary-data-based Mendelian Randomization (SMR) analyses with the associated heterogeneity in dependent instruments (HEIDI) test (8). We found increased expression of miR-1908-5p to be associated with bipolar disorder (*P*-SMR = 5.78 × 10^−7^; Fig. 3), depressive symptoms (*P*-SMR = 1.19 × 10^−5^), irritability (*P*-SMR = 1.19 × 10^−5^), increased right cerebellum exterior volume (*P*-SMR = 3.85 × 10^−5^) and increased sleep duration (*P*-SMR = 9.51 × 10^−6^), but not cognitive performance, intelligence or left cerebellum exterior volume, at the Bonferroni-corrected threshold (Supplementary Material, Fig. S2). The HEIDI test was non-significant (*P* > 0.05) for all five traits (Supplementary Material, Fig. S2), indicating that these associations are unlikely to be driven by linkage disequilibrium between the eQTL and independent variants influencing these phenotypes.

### Predicted targets of miR-1908-5p expressed in human fetal brain are enriched for genetic association with bipolar disorder

We sought independent evidence for a role of prenatal miR-1908-5p function in bipolar disorder, depressive symptoms, irritability, exterior cerebellum volume and sleep duration by testing whether genes that are potentially regulated by miR-1908-5p are also enriched for genetic association with each trait. Predicted targets were determined using TargetScan 8.0 (https://www.targetscan.org/vert_80/), a miRNA target prediction tool that searches for the presence of conserved miRNA response elements (8mer, 7mer and 6mer sites) in the 3’-UTR of mRNAs matching the seed region of the specified miRNA. Genes predicted to be regulated by miR-1908-5p were enriched for Gene Ontology terms relating to neuronal development, synaptic signalling and regulation of transcription (Supplementary Material, Table S4). A MAGMA (Multi-marker Analysis of GenoMic Annotation) competitive gene-set association analysis (40) was then performed on all predicted mRNA targets detected in second trimester fetal brain (18) using the GWAS statistics for each trait (27,34,36,38,39) and a background of all annotated genes expressed in fetal brain (18) (Table 2). Predicted gene targets of miR-1908-5p were found to be significantly enriched for genetic association with bipolar disorder (Bonferroni-corrected *P* = 0.013), consistent with altered expression of miR-1908-5p in the developing brain acting as a risk factor for the condition. We did not find SNP rs174561 to be a *trans*-eQTL for any of the predicted mRNA targets of miR-1908-5p at an FDR < 0.05 in fetal brain (18) (Supplementary Material, Table S5), although we note that *trans*-eQTL are typically of smaller effect than *cis*-eQTL (41) and will therefore require larger sample sizes for their discovery.

**Table 2:** Enrichment of genetic association for five brain-related traits in predicted gene targets of miR-1908-5p expressed in second trimester fetal brain

Trait	Beta	Beta SD	SE	*P*	*P*_corr_
Bipolar disorder	0.066	0.022	0.023	0.0025	0.013
Sleep duration	0.034	0.011	0.023	0.070	0.35
Irritability	-0.0071	-0.0024	0.021	0.63	1
Depressive symptoms	0.024	0.0081	0.025	0.16	0.8
Right cerebellum exterior	0.039	0.013	0.020	0.028	0.14

Analyses performed using MAGMA (40) and GWAS statistics for each trait (27,34,36,38,39), with a background of all annotated genes expressed in fetal brain (18). Beta = regression coefficient of the predicted mRNA target gene-set, BETA SD = semi-standardized regression coefficient, SE = the standard error of the regression coefficient, *P*_corr_ = *P*-value after Bonferroni correction for five tests.

## Discussion

We have identified genetic variants associated with the expression of 30 miRNA in the developing human brain at an FDR < 0.05, of which 23 are novel findings. Identified miR-eQTL are typically within 100 kb of the genomic location of the associated mature miRNA sequence. Most influence miRNA that are located within host mRNA gene exonic or intronic sequence but are generally not associated with host mRNA expression in fetal brain. We identified one miRNA eQTL for which there is strong evidence for association with bipolar disorder and find evidence that increased expression of its target, miR-1908-5p, also influences depressive symptoms, irritability, exterior cerebellum volume and sleep duration in the general population.

The recent study of Lafferty and colleagues (17) is the only other miR-eQTL analysis performed on human fetal brain published to date. That study was performed on cortical brain tissue from 212 fetuses aged between 14 and 21 gestational weeks, identifying high-confidence (FDR < 0.05) eQTL for 70 miRNA. As noted by Lafferty et al, the proportion of miRNA identified as being influenced by *cis-*eQTL is considerably lower than the proportion of mRNA with eQTL identified in similar sized fetal brain collections (18,19), potentially reflecting greater selection pressure on miRNA expression, given the number of genes they regulate. However, as most of the high-confidence miR-eQTL identified in this and the previous study were unique to each, it appears that current sample sizes provide low power to detect miR-eQTL operating in fetal brain at typical effect sizes.

The seven miRNA with high-confidence (FDR < 0.05) eQTL detected in both this and the study of Lafferty et al. (17) include miR-4707-3p. The most significant eQTL for miR-4707-3p in the present study was rs2273626, located within the seed sequence of the mature miR-4707-3p and predicted to alter pri-miRNA hairpin secondary structure as well as miRNA targeting. The index eQTL for miR-4707-3p in the study of Lafferty and colleagues (17) was rs4981455, a SNP approximately 3.7 kb away from genomic sequence encoding the mature miR-4707, which the authors note is in high linkage disequilibrium (*r*^2^ > 0.99) with rs2273626. We found that the A-allele of rs2273626 is associated with lower miR-4707-3p expression in the second trimester fetal brain, consistent with the other study (17), which also reported lower allele-specific expression of this SNP. Although we found no strong association between rs2273626 and any of the 11 tested developmental, neurological or psychiatric conditions, Lafferty and colleagues report colocalisation between miR-4707-3p eQTL and variants associated with educational attainment and indices of brain size (17).

Only one of the 30 high-confidence miRNA eQTL operating in fetal brain detected in this study was found to be associated with any of the 11 initially screened developmental, neurological or psychiatric phenotypes at the Bonferroni-corrected *P*-value threshold (*P* < 1.5 × 10^−4^). This SNP (rs174561), associated with both miR-1908-5p expression and, at genome-wide significance, bipolar disorder, is located within the pri-miRNA hairpin of miR-1908 and is predicted to alter its secondary structure. Although this SNP (or one in high linkage disequilibrium with it) was not one of the high-confidence miRNA eQTL identified by Lafferty et al. (17), it has been reported as the most significant eQTL for miR-1908-5p in blood (42). As previously noted (43,44), the C-allele of rs174561 (associated with higher expression of miR-1908-5p in the present study) is predicted to produce a pri-miRNA hairpin that is more thermostable than the alternative T-allele. The *MIR1908* gene (which gives rise to both miR-1908-5p and miR-1908-3p from different arms of its pri-miRNA hairpin) has been highlighted as a potential risk factor for bipolar disorder in gene-based association analyses of GWAS data (44,45). The present findings add to this literature by implicating increased expression of miR-1908-5p in the developing brain as a plausible mechanism mediating genetic association between this locus and the disorder.

We found that predicted mRNA targets of miR-1908-5p that are expressed in fetal brain are enriched for genetic association with bipolar disorder, providing independent evidence that prenatal expression of this miRNA acts as a risk factor for the condition. An enrichment of genetic association with bipolar disorder has also been observed in predicted miR-1908-5p mRNA targets that are expressed in adult human brain (*P_uncorrected_* = 0.012) (44), although the significance of this finding did not survive correction for multiple testing in that study. Validated mRNA targets of miR-1908-5p include the synaptic genes *CLSTN1*, *DLGAP4*, *GRIN1*, *GRM4* and *STX1A* (46), which are expressed in both the pre- and post- natal brain. To our knowledge, rs174561 has not been reported as a high-confidence eQTL for miR-1908-5p in adult brain tissue. However, given that this SNP plausibly impacts miR-1908-5p expression through direct effects on its pri-miRNA hairpin structure, it might be expected to be functional in all tissues in which it is expressed.

As well as its association with bipolar disorder, we found evidence that increased expression of miR-1908-5p in human fetal brain is associated with increased depressive symptoms, irritability, right cerebellum exterior volume and sleep duration in the general population. In addition, we were made aware during review that SNP rs174561 is a genome-wide significant index SNP for depression (*P* = 2.54 × 10^−12^) in a recent cross-ancestry GWAS, currently available as a preprint (47). Depressive symptoms and irritability are core features of bipolar disorder, while sleep disturbances, including hypersomnia, are commonly seen in the condition (48). However, in contrast to our findings for bipolar disorder, we did not find compelling evidence that predicted mRNA targets of miR-1908-5p expressed in fetal brain are enriched for genetic association with these other traits. Aside from potential differences in statistical power between the different GWAS tested, this might suggest that miR-1908-5p expression in the developing brain is less relevant to these traits, or that its association is mediated by a smaller number of targets than it is for bipolar disorder. Validation of predicted miR-1908-5p targets in different cell types and stages of development could shed light on this issue.

A limitation of this and prior eQTL studies of miRNA expression in human brain is that measures were performed on bulk tissue, making it difficult to discern which cell populations *cis*-regulatory polymorphism are operating, and potentially obscuring cell-specific effects. Single nuclei RNA sequencing is likely to prove useful for mapping cell-specific eQTL associated with (pri-/pre-) miRNA expression in the human brain and delineating RNA targets expressed in those cell types. Increases in sample size should reveal additional miRNA eQTL operating during brain development, as has been demonstrated in larger eQTL studies of mRNA expression performed in adult (49,50) and fetal (51) brain. Such studies may clarify the mechanisms by which altered expression of miR-1908-5p confers risk to bipolar disorder, as well as indicate other miRNA with roles in neuropsychiatric illness.

## Materials and Methods

### Samples

Human fetal brain tissue from elective terminations of pregnancy (12–20 post-conception weeks) was provided by the MRC-Wellcome Trust Human Developmental Biology Resource (HDBR) (http://www.hdbr.org). Ethical approval for the collection and distribution of fetal material for scientific research was granted to the HDBR by the Royal Free Hospital Research Ethics Committee and North East-Newcastle and North Tyneside Research Ethics Committee. Fetal age was determined by the HDBR through foot length and knee-to-heel length measurements and sex was determined by heterozygosity for X-chromosome markers in females. Samples were of normal karyotype. No other information was available. Total RNA and genomic DNA had been extracted from the majority of samples (obtained as frozen, undissected brain tissue) for previous studies (18,52,53) using Tri-Reagent (Thermo Fisher Scientific) and phenol-chloroform, respectively. The same methodology was employed to extract total RNA and genomic DNA from a further 19 second trimester fetal brain samples specifically for this study. Following exclusion of samples that failed genotyping or RNA sequencing quality control, 112 independent samples (51 female, 61 male) were available for eQTL analysis.

### Genotyping

Genome-wide genotype data were available for all except 19 of the assayed samples through previous studies (18,53). The additional 19 samples were also genotyped using the Infinium OmniExpress-24 BeadChip array (Illumina), which directly captures approximately 710 000 single nucleotide polymorphisms (SNPs). Samples with > 5% missing SNPs or anomalous heterozygosity were excluded. SNPs missing genotypes in > 5% of samples or with minor allele frequencies < 0.01 were removed, along with A/T and G/C SNPs with minor allele frequencies > 0.4. The SNP strand and ref/alt assignment were updated to match the Haplotype Reference Consortium (HRC) version 1.1 (54), and SNPs for which the minor allele frequency differed by > 0.2 from the HRC version were removed. Additional SNPs were imputed from the HRC panel using minimac3 and Eagle v2.3 phasing through the Michigan Imputation Server (https://imputationserver.sph.umich.edu/index.html).

SNPs were annotated with rsID numbers from dbSNP v149 and converted to GRCh38 coordinates using CrossMap (55) using chain files from the UCSC Genome Browser (https://genome.ucsc.edu/). checkVCF (https://github.com/zhanxw/checkVCF) was employed to identify and remove non-SNP positions and duplicate sites. SNPs with an imputation *r*^2^ less than 0.8, minor allele frequency less than 0.05, or Hardy–Weinberg equilibrium violation *P*-values < 1 × 10^−4^ in the entire sample were also excluded.

### Small RNA sequencing

Small RNA libraries were constructed from 1 microgram of DNAse-treated total RNA from each sample using TruSeq Small RNA Library Preparation kits (Illumina) in accordance with manufacturer guidelines. Following quantification through qPCR, libraries were pooled in equimolar amounts and sequenced on an Illumina HiSeq 4000 system. The resulting 50-bp single-end sequencing reads were analysed according to a recently published protocol for miRNA sequencing studies (56). Quality control of sequencing was performed using FastQC and MultiQC, and adapters trimmed using trimmomatic (57). Samples with low-quality sequencing data and/or < 2 million sequencing reads were excluded. Trimmed reads were filtered to exclude those containing < 16 or > 31 nucleotides using Cutadapt v3.2. Filtered reads were aligned using Bowtie1 in a two-step approach, as documented by Potla and colleagues (56). First, reads were aligned to mature miRNA reads in miRbase v22 (ref. 2) allowing no mismatches. Subsequently, the unaligned reads from the first alignment were aligned to the GRCh38 reference genome allowing for one mismatch between the miRNA reads and the reference genome. Mapped reads were visualized using the Integrative Genomics Viewer (58), and mapping quality was ascertained using Qualimap (59). Aligned reads were quantified as raw counts using SAMtools idxstats for miRbase aligned counts and featurecounts (60) for genome-mapped reads. Raw miRNA counts derived from aligning each miRNA to miRbase and to the GRCh38 reference genome were then merged, and miRNAs with 0 counts in more than 90% of the samples were eliminated. A total of 1449 miRNAs were detected. Reads for each miRNA were normalized using the trimmed mean of M-values (TMM) method and transformed into log2 CPM +1 using edgeR (61).

### miRNA eQTL analysis

miRNA *cis-*eQTL analyses were carried out by linear regression using FastQTL (21) in 112 samples with both genotype and small RNA sequencing measures that passed QC. TMM normalized miRNA expression measures were corrected for age, sex, RIN, sequencing batch, the first three genotype principal components, average read quality, % duplicates after filtering, % GC content after filtering, average read length after filtering (bp), total amount of reads after filtering, % of mapped reads to miRNAs and 10 hidden confounders estimated through PEER (62). The residuals were used to test for association between the expression of each miRNA and SNPs located within 500 kb either side of the first base of its mature sequence. An FDR for each eQTL was calculated by first correcting *P*-values for the number of SNPs tested per miRNA through estimation of a beta distribution using a minimum of 1000 permutations (maximum 10 000), and then correcting these *P*-values for the number of miRNA tested using Storey’s *q*-value method (63). In keeping with previous eQTL studies of human fetal brain (17–19), we primarily report *cis*-eQTL with FDR < 0.05.

### Characterisation of identified miRNA eQTL

The genomic position of the most significant *cis-*eQTL (FDR < 0.05) in relation to its differentially regulated miRNA was determined using FastQTL (21) and plotted using R4.1.2. Linkage disequilibrium between the most significant eQTL (FDR < 0.05) for each miRNA identified in this study and those reported by Lafferty and colleagues (17) (where the two studies did not implicate the same SNP) was calculated using LDPair (https://analysistools.cancer.gov/LDlink/?tab=ldpair). Potential direct effects of top eQTL located within the pri-miRNA hairpin sequence on hairpin thermodynamic stability were explored using the software RNAfold (64), applying the default parameters in the miRNASNP-v3 database (http://bioinfo.life.hust.edu.cn/miRNASNP/) (65). The plot of the genotypic association of miR-eQTL with miRNA expression was generated using the R package ggstatsplot (66).

### Association between detected miRNA eQTL and brain traits

We initially screened GWAS summary data for 11 neurodevelopmental, neurological or psychiatric conditions for evidence of association with the most significant eQTL for the 30 variably *cis*-regulated miRNA identified in this study. These were Alzheimer’s disease (22), anorexia (23), anxiety disorder (24), attention-deficit hyperactivity disorder (25), autism (26), bipolar disorder (27), depression (28), obsessive-compulsive disorder (29), post-traumatic stress disorder (30), schizophrenia (31) and Tourette Syndrome (32). GWAS summary data were obtained from the Psychiatric Genomics Consortium website repository (https://pgc.unc.edu/) or, in the case of Alzheimer’s disease (22), the GWAS catalogue (https://www.ebi.ac.uk/gwas/studies/GCST90027158). For top miR-1908-5p eQTL rs174561, we additionally screened 836 psychiatric, neurological and cognitive traits using the Atlas of GWAS Summary Statistics (https://atlas.ctglab.nl/; ref. 33). Any findings surpassing the Bonferroni-corrected *P*-value thresholds (*P* < 1.5 × 10^−4^ to account the top eQTL for 30 miRNA across 11 traits, or *P* < 6 × 10^−5^ to account for single eQTL rs174561 across 836 traits) were also analysed by SMR and HEIDI (8) as a more formal test of joint associations and to distinguish pleiotropy from associations driven by genetic linkage. For these analyses, SNPs with allele frequency difference > 0.2 between any two datasets (eQTL summary data, GWAS summary data or 1000 Genomes EUR samples as the linkage disequilibrium reference) were removed. For SMR, we set the same Bonferroni-corrected *P*-value thresholds as used for the initial screen by which the trait was highlighted (i.e. *P* < 1.5 × 10^−4^ for bipolar disorder and *P* < 6 × 10^−5^ for the other brain traits). The SMR locus plots were generated using R4.1.2, with the thresholds *P*-SMR = 1 and *P*-HEIDI = 0, as suggested by the authors (8). The HEIDI test was performed after removing SNPs in very strong linkage disequilibrium (*r*^2^ > 0.9) or absent linkage disequilibrium (*r*^2^ < 0.05) with the top associated miR-eQTL. In the HEIDI test, rejection of the null hypothesis indicates that the observed association could be due to two distinct genetic variants in linkage disequilibrium with each other. We set this at *P* < 0.05, consistent with the original paper (8).

### Testing predicted targets of miR-1908-5p for genetic association with bipolar disorder and other traits

Predicted mRNA targets of miR-1908-5p were retrieved from TargetScan 8.0 (67). Target gene names were converted into Entrez IDs using the Biomart package (68). Predicted targets of miR-1908-5p were tested for enrichment in Biological Process Gene Ontology terms using g:Profiler (69). MAGMA (40) was used to test enrichment of trait-associated genetic variation in the gene set comprising all predicted targets of miR-1908-5p detected in fetal brain (18) based on GWAS summary statistics for each trait and using a background of all genes expressed in fetal brain. SNPs were annotated to a gene if they were located within a window extending 35 kb upstream and 10 kb downstream of its transcribed region.

### Testing of rs174561 as a *trans*-eQTL for predicted mRNA targets of miR-1908-5p in prenatal human brain

Genotypes at rs174561 and genome-wide measures of RNA expression (corrected for covariates) derived from 120 human brain samples from the second trimester of gestation were obtained from our mRNA-based eQTL study in fetal brain (18). *Trans*-eQTL analysis was performed by linear regression using Matrix eQTL (70), restricted to SNP rs174561 and the 2150 miR-1908-5p targets predicted by TargetScan 8.0 (67) that were found to be expressed in fetal brain (18). FDR was calculated by the Matrix eQTL package (70).

## Supplementary Material

Toste_Supplementary_Figures_revised_ddad118

Toste_Supplementary_Tables_revised_ddad118

## Data Availability

Summary statistics for miRNA expression, covariates and all eQTL are provided through a publicly accessible figshare repository: https://doi.org/10.6084/m9.figshare.22674109.v1.
