# Supplementary material for: Mapping microRNA expression quantitative trait loci in the prenatal human brain implicates miR-1908-5p expression in bipolar disorder and other brain-related traits
Source: Hum Mol Genet. 2023 Jul 20;32(20):2941–9. doi: 10.1093/hmg/ddad118 (PMC10549788; doi:10.1093/hmg/ddad118)
Supplement: Toste_Supplementary_Figures_revised_ddad118 [file toste_supplementary_figures_revised_ddad118.docx]

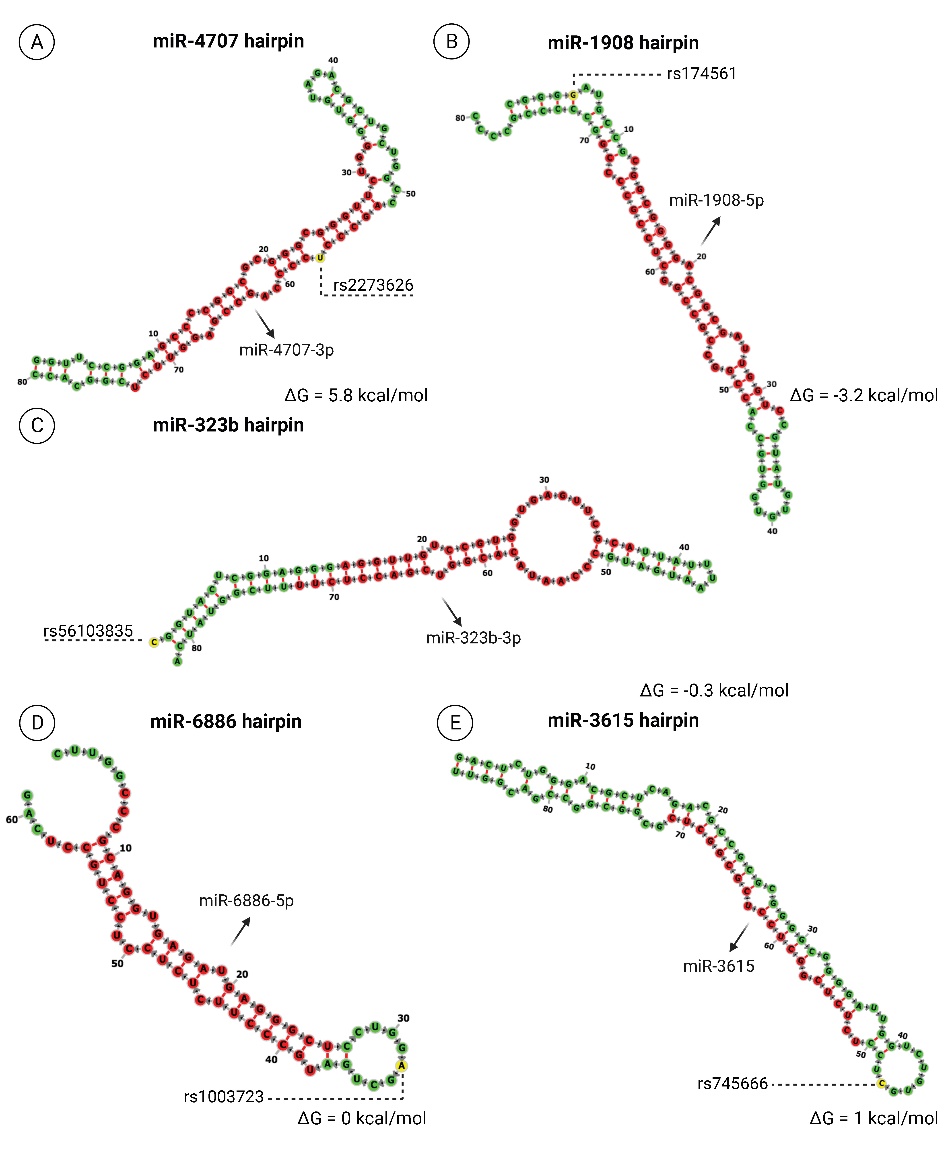


**Supplementary Figure 1.** Predicted effects of top miR-eQTL located in pri-miRNA hairpin sequence on miRNA hairpin secondary structure using the software RNAfold (Lorenz et al, 2011) and default parameters in the miRNASNP-v3 database (http://bioinfo.life.hust.edu.cn/miRNASNP/) (Liu et al, 2021). Pri-miRNA hairpins were retrieved from the miRNASNP-v3 database (Liu et al, 2021). Red = mature miRNA sequence; Green = pri-miRNA sequence not in mature miRNA; Yellow = miR-eQTL SNP position. **A)** The miR-4707 hairpin gives rise to 2 mature miRNAs: miR-4707-5p and miR-4707-3p. miR- 4707-3p eQTL SNP rs2273626 is in the seed region of miR-4707-3p and is predicted to alter minimum free energy of the hairpin. **B)** The miR-1908 hairpin can give rise to 2 mature miRNAs: miR-1908-5p and miR-1908-3p. miR-1908-5p eQTL SNP rs174561 is at base position 5 of the miR-1908 hairpin and is predicted to alter its minimum free energy. **C)** The paternally imprinted miR-323b hairpin can give rise to 2 mature miRNAs: miR-323b-5p and miR-323b-3p. miR-323b-3p eQTL SNP rs56103835 modifies the regulatory basal UG motif of the miR-323b hairpin and is predicted to result in a small change in minimum free energy. **D)** The miR-6886 hairpin can give rise to 2 mature miRNAs: miR-6886-5p and miR-6886-3p. miR-6886-5p eQTL SNP rs1003727 is in the apical loop of the miR-6886 hairpin and is not predicted to alter minimum free energy. **E)** The miR-3615 hairpin gives rise to miR-3615. eQTL SNP rs745666 is located in the apical loop of the hairpin and is predicted to alter minimum free energy.

**References**

Lorenz, R., Bernhart, S. H., Höner Zu Siederdissen, C., Tafer, H., Flamm, C., Stadler, P. F. and Hofacker, I. L. (2011) ViennaRNA Package 2.0. *Algorithms Mol. Biol*., **6**, 26.

Liu, C. J., Fu, X., Xia, M., Zhang, Q., Gu, Z. and Guo, A. Y. (2021) miRNASNP-v3: a comprehensive database for SNPs and disease-related variations in miRNAs and miRNA targets. *Nucleic Acids Res*., **49**, D1276–D1281.


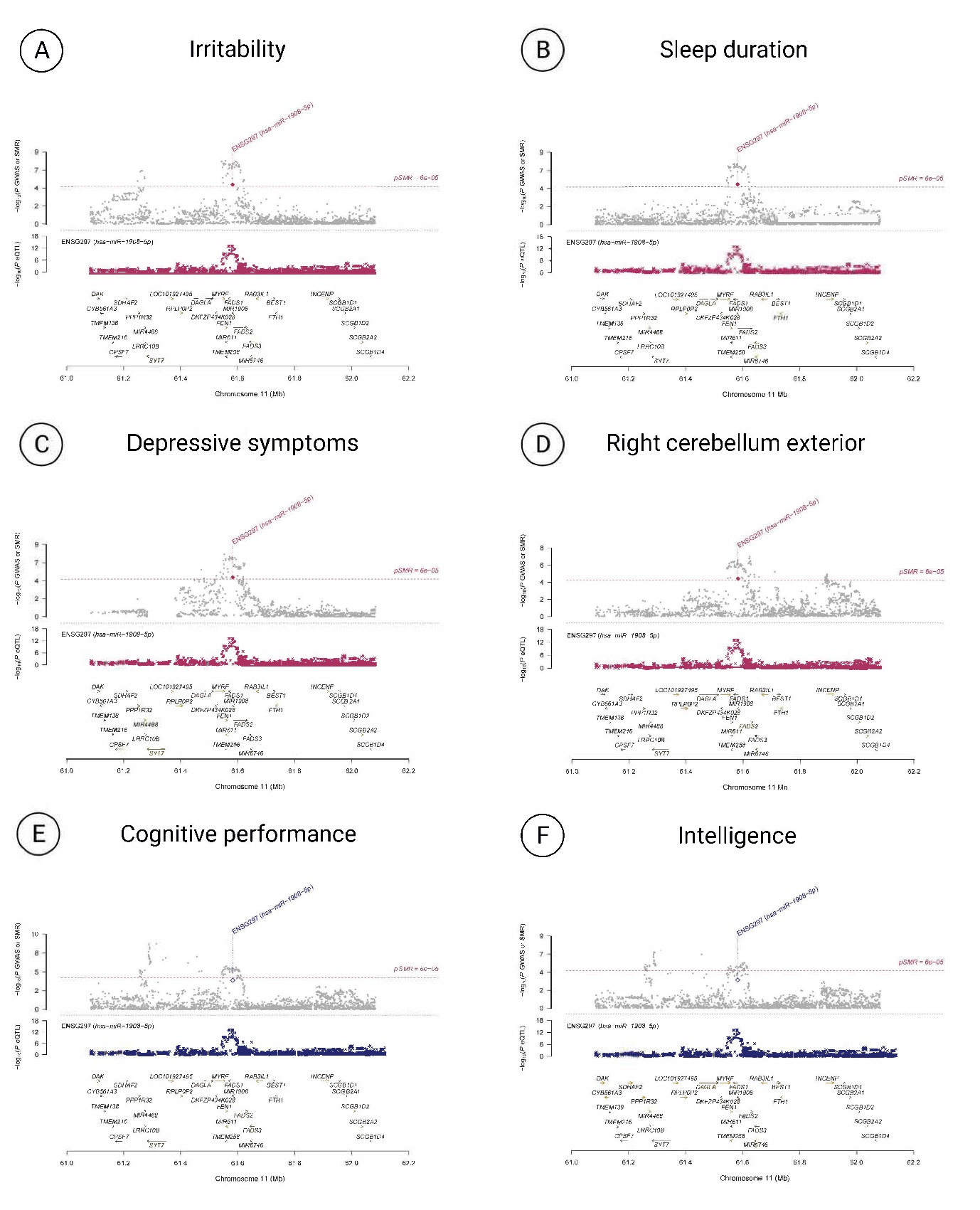


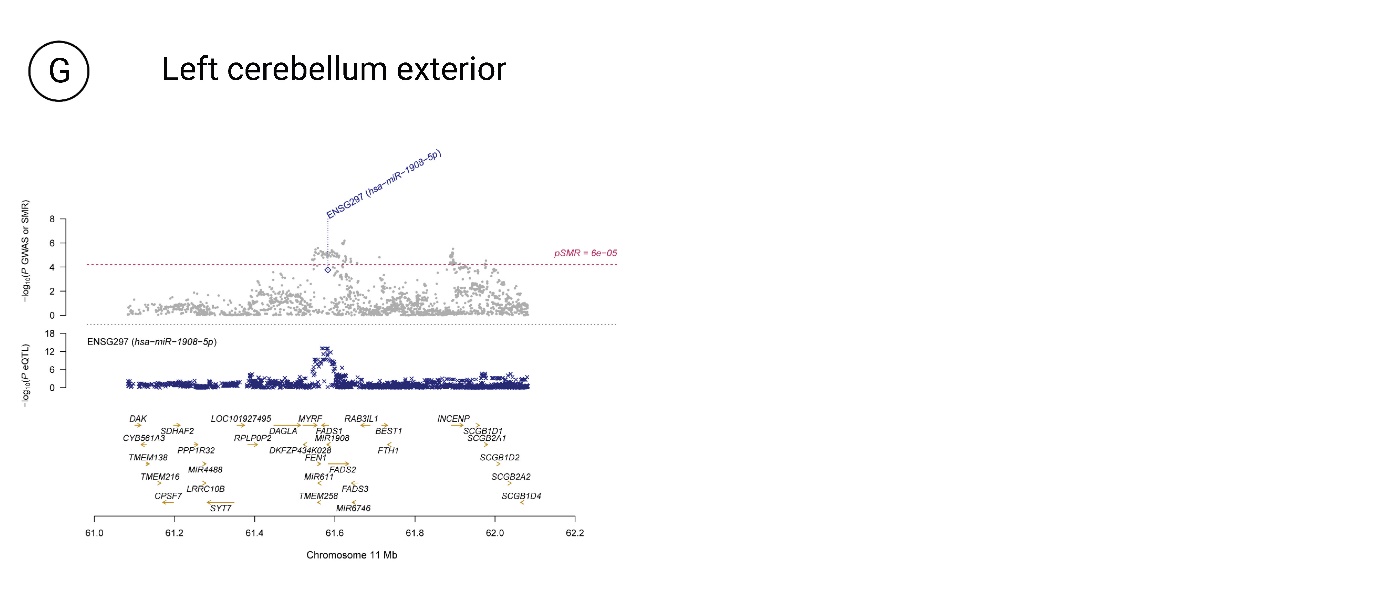


**Supplementary Figure 2.** SMR analysis of genetic variants associated with miR-1908-5p expression in relation to genetic variants influencing (A) irritability (Nagel et al, 2018), (B) sleep duration (Dashti et al, 2019), (C) depressive symptoms (Baselmans et al, 2019), (D) right exterior cerebellum volume (Zhao et al, 2019), (E) cognitive performance (Lee et al, 2018), (F) intelligence (Savage et al, 2018) and (G) left exterior cerebellum volume (Zhao et al, 2019) in general population-based samples. In each panel, the top plot shows log_10_ *P*-values for association between SNPs at the chromosome 11 (61-62Mb) locus and each trait. The diamond represents the -log_10_ *P*-value of miR-1908-5p expression in the SMR test and the dashed line indicates the Bonferroni-corrected *P*-value threshold for the initial screen of 836 traits through the Atlas of GWAS Summary Statistics (Watanabe et al, 2019). The bottom plot in each panel shows the -log_10_ *P*-values for associations between SNPs at the chromosome 11 (61-62Mb) locus and miR-1908-5p expression in fetal brain in this study. Those in red (A-D) indicate traits where the SMR test passes the Bonferroni-corrected *P*-value threshold. Genomic coordinates are hg19.

**References**

Nagel, M., Watanabe, K., Stringer, S., Posthuma, D., van der Sluis, S. (2018) Item-level analyses reveal genetic heterogeneity in neuroticism. *Nat. Commun*., **9**, 905.

Dashti, H. S., Jones, S. E., Wood, A. R., Lane, J. M., van Hees, V. T., Wang, H., Rhodes, J. A., Song, Y., Patel, K., Anderson, S. G., et al (2019) Genome-wide association study identifies genetic loci for self-reported habitual sleep duration supported by accelerometer-derived estimates. *Nat. Commun*., **10**, 1100.

Baselmans, B. M. L., Jansen, R., Ip, H. F., van Dongen, J., Abdellaoui, A., van de Weijer, M. P., Bao, Y., Smart, M., Kumari, M., Willemsen, G., et al (2019) Multivariate genome-wide analyses of the well-being spectrum. *Nat. Genet*., **51**, 445–451.

Zhao, B., Luo, T., Li, T., Li, Y., Zhang, J., Shan, Y., Wang, X., Yang, L., Zhou, F., Zhu, Z.,et al. (2019) Genome-wide association analysis of 19,629 individuals identifies variants influencing regional brain volumes and refines their genetic co-architecture with cognitive and mental health traits. *Nat. Genet*., **51**, 1637–1644.

Lee, J. J., Wedow, R., Okbay, A., Kong, E., Maghzian, O., Zacher, M., Nguyen-Viet, T. A., Bowers, P., Sidorenko, J., Karlsson Linnér, R., et al (2018) Gene discovery and polygenic prediction from a genome-wide association study of educational attainment in 1.1 million individuals. *Nat. Genet*., **50**, 1112–1121.

Savage, J. E., Jansen, P. R., Stringer, S., Watanabe, K., Bryois, J., de Leeuw, C. A., Nagel, M., Awasthi, S., Barr, P. B., Coleman, J. R. I., et al (2018) Genome-wide association meta-analysis in 269,867 individuals identifies new genetic and functional links to intelligence. *Nat. Genet*., **50**, 912–919.

Watanabe, K., Stringer, S., Frei, O., Umićević Mirkov, M., de Leeuw, C., Polderman, T. J. C., van der Sluis, S., Andreassen, O. A., Neale, B. M. and Posthuma, D. (2019) A global overview of pleiotropy and genetic architecture in complex traits. *Nat. Genet*., **51**, 1339–134.
