# Supplementary material for: Mapping microRNA expression quantitative trait loci in the prenatal human brain implicates miR-1908-5p expression in bipolar disorder and other brain-related traits
Source: Hum Mol Genet. 2023 Jul 20;32(20):2941–9. doi: 10.1093/hmg/ddad118 (PMC10549788; doi:10.1093/hmg/ddad118)
Supplement: Toste_Supplementary_Tables_revised_ddad118 [file toste_supplementary_tables_revised_ddad118.docx]

| **miRNA** | **miR-eQTL** | **Host Gene** | **q-value of**  **miR-eQTL** | **p-value (nominal) of host gene eQTL** |
| --- | --- | --- | --- | --- |
| miR-5683 | rs7769202 | F13A1 | 8.91E-13 | 0.29 |
| miR-4707-3p | rs2273626 | HAUS4 | 3.21E-12 | 2.20E-04 |
| miR-544b | rs3821536 | UMPS | 1.80E-08 | 0.25 |
| miR-6868-3p | rs2243486 | EXOC7 | 1.80E-08 | 0.28 |
| miR-1908-5p | rs174561 | FADS1 | 9.27E-08 | 0.071 |
| miR-4326 | rs7263455 | ARFGAP1 | 5.85E-07 | 0.22 |
| miR-3615 | rs745666 | SLC9A3R1 | 6.43E-07 | 0.81 |
| miR-548ba | rs2140551 | FSHR | 7.40E-07 | - |
| miR-1287-5p | rs942803 | PYROXD2 | 1.63E-05 | 0.061 |
| miR-3117-3p | rs1925342 | SGIP1 | 5.98E-05 | 0.12 |
| miR-4467 | rs6971245 | LRWD1 | 6.95E-05 | 0.75 |
| miR-4803 | rs1561401 | MAP1B | 2.19E-04 | 0.64 |
| miR-7854-3p | rs2927318 | CMIP | 2.43E-04 | 0.4 |
| miR-548at-5p | rs11653901 | ATP6V0A1 | 1.05E-03 | 0.15 |
| miR-3161 | rs74236456 | PTPRJ | 1.23E-03 | 0.11 |
| miR-3125 | rs6717278 | TRIB2 | 2.07E-03 | 0.96 |
| miR-3176 | rs116698525 | CAPN15 | 2.66E-03 | 0.59 |
| miR-4761-3p | rs4680 | COMT | 3.25E-03 | 0.23 |
| miR-641 | rs41275750 | AKT2 | 0.016 | 0.45 |
| miR-6886-5p | rs1003723 | LDLR | 0.031 | 0.45 |
| miR-6826-3p | rs6788178 | COPG1 | 0.039 | 0.83 |
| miR-3938 | rs55852613 | ERC2 | 0.041 | 0.27 |

**Supplementary Table 1**. *P*-values for high confidence miR-eQTL (FDR < 0.05) as eQTL for their host genes in human fetal brain (O’Brien et al, 2018).

**Reference:** O'Brien, H. E., Hannon, E., Hill, M. J., Toste, C. C., Robertson, M. J., Morgan, J. E., McLaughlin, G., Lewis, C. M., Schalkwyk, L. C., Hall, L. S., et al (2018) Expression quantitative trait loci in the developing human brain and their enrichment in neuropsychiatric disorders. *Genome Biol.*, **19**, 194.

| **SNP ID** | **miRNA** | **AD**  **(2022)** | **Anorexia (2019)** | **Anxiety**  **(2016)** | **ADHD**  **(2019)** | **AUT**  **(2019)** | **BD**  **(2021)** | **Dep**  **(2019)** | **OCD**  **(2018)** | **PTSD**  **(2019)** | **SZ**  **(2022)** | **TS**  **(2019)** |
| --- | --- | --- | --- | --- | --- | --- | --- | --- | --- | --- | --- | --- |
| **rs7769202** | miR-5683 | 0.14 | 0.38 | 0.12 | _ | 0.04 | 0.78 | 0.02 | 0.55 | 0.33 | 0.85 | 0.28 |
| **rs2273626** | miR-4707-3p | 0.03 | 3.66E-03 | 0.17 | 0.10 | 1.00 | 0.07 | 0.12 | 0.26 | 0.52 | 0.04 | 0.10 |
| **rs3821536** | miR-544b | 0.32 | 0.31 | 0.22 | 0.90 | 0.83 | 0.19 | 0.78 | 0.06 | 0.17 | 0.12 | 0.97 |
| **rs2243486** | miR-6868-3p | 0.74 | 0.62 | 0.42 | 0.08 | 0.19 | 0.46 | 0.26 | 0.20 | 0.29 | 0.98 | 0.92 |
| **rs10862209** | miR-618 | 0.25 | 0.42 | 0.07 | 0.51 | 0.51 | 0.43 | 0.42 | 0.09 | 0.23 | 0.39 | 0.07 |
| **rs174561** | miR-1908-5p | 0.67 | 0.89 | 0.48 | 0.85 | 0.80 | **1.83E-11** | 1.35E-03 | 0.69 | 0.78 | 0.03 | 0.32 |
| **rs7263455** | miR-4326 | 0.89 | _ | 0.29 | _ | 0.09 | 0.24 | _ | 0.42 | 0.50 | 0.21 | 0.89 |
| **rs745666** | miR-3615 | 0.85 | _ | 0.13 | _ | 0.47 | 0.90 | _ | 0.12 | 0.15 | 0.77 | 0.22 |
| **rs2140551** | miR-548ba | 0.22 | 0.83 | 0.40 | 0.77 | 0.10 | 0.65 | 0.43 | 0.43 | 0.50 | 0.02 | 0.74 |
| **rs72641631** | miR-1269a | 0.62 | 0.51 | 0.70 | 0.79 | 0.67 | 0.44 | 0.88 | 0.81 | 0.35 | 0.80 | 0.08 |
| **rs942803** | miR-1287-5p | 0.86 | 0.31 | 0.23 | 0.89 | 0.33 | 0.03 | 0.33 | 0.84 | 0.54 | 0.56 | 0.74 |
| **rs1925342** | miR-3117-3p | 0.27 | 0.25 | 0.84 | 0.86 | 0.19 | 0.22 | 0.03 | 0.88 | 0.47 | 0.97 | 0.99 |
| **rs6971245** | miR-4467 | 0.86 | 0.86 | 0.75 | 0.65 | 0.04 | 0.14 | 0.78 | 0.25 | 0.71 | 0.59 | 0.71 |
| **rs1561401** | miR-4803 | 0.71 | 0.49 | 0.50 | 0.95 | 0.87 | 0.99 | 0.18 | 0.27 | 0.10 | 0.98 | 0.41 |
| **rs56103835** | miR-323b-3p | 0.48 | 0.90 | 0.26 | 0.77 | 0.99 | 0.41 | 0.01 | 0.16 | 0.36 | 0.11 | 0.58 |
| **rs2927318** | miR-7854-3p | 0.6 | 0.86 | 0.88 | 0.44 | 0.54 | 0.72 | 0.99 | 0.64 | 0.22 | 0.66 | 0.53 |
| **rs7006762** | miR-4662a-5p | 0.73 | 0.28 | 0.84 | 0.97 | 0.89 | 0.07 | 0.53 | 0.16 | 0.66 | 0.59 | 0.31 |
| **rs11653901** | miR-548at-5p | 0.66 | 0.23 | 0.62 | 0.28 | 0.28 | 0.27 | 0.78 | 0.18 | 0.09 | 6.72E-04 | 0.97 |
| **rs74236456** | miR-3161 | 0.56 | 0.1 | 0.90 | 0.38 | 0.01 | 0.06 | 0.14 | 0.37 | 0.54 | 0.98 | 0.11 |
| **rs6717278** | miR-3125 | 1.64E-03 | 0.59 | 0.16 | 0.23 | 0.57 | 0.60 | 0.97 | 0.89 | 0.82 | 0.03 | 0.53 |
| **rs116698525** | miR-3176 | 0.44 | 0.92 | 0.37 | _ | 0.58 | 8.45E-03 | 6.23E-03 | 0.29 | 0.89 | 0.04 | 0.18 |
| **rs28576121** | miR-1270 | 0.79 | _ | 0.76 | 0.21 | 0.15 | 0.57 | 0.43 | 0.08 | 0.20 | 0.55 | 0.09 |
| **rs4680** | miR-4761-3p | 0.95 | 0.94 | 0.94 | 0.98 | 0.59 | 0.48 | 0.67 | 0.92 | 0.11 | 0.02 | 0.09 |
| **rs41275750** | miR-641 | 0.65 | 0.36 | 0.73 | 0.05 | 0.16 | 0.38 | 0.31 | 0.25 | 0.28 | 0.90 | 0.65 |
| **rs709777** | miR-4423-5p | 0.84 | 0.86 | 0.93 | 0.04 | 0.27 | 0.59 | 0.51 | 0.09 | 0.21 | 0.82 | 0.89 |
| **rs1003723** | miR-6886-5p | 1.92E-02 | 0.85 | 0.04 | 0.27 | 0.09 | 0.87 | 0.09 | 0.56 | 0.11 | 0.20 | 0.18 |
| **rs11101657** | miR-202-5p | 0.85 | _ | _ | _ | _ | 0.16 | _ | 0.90 | 0.14 | 0.14 | 9.60E-03 |
| **rs112622797** | miR-6840-5p | 0.55 | 0.09 | 0.64 | 0.34 | 0.32 | 0.60 | 0.38 | 0.90 | 0.89 | 0.15 | 0.52 |
| **rs6788178** | miR-6826-3p | 8.30E-03 | 0.24 | 0.26 | 0.09 | 0.51 | 0.23 | 0.73 | 0.03 | 0.11 | 2.51E-04 | 0.86 |
| **rs55852613** | miR-3938 | 0.41 | 0.26 | 0.18 | 0.24 | 0.29 | 0.70 | 0.13 | 0.46 | 0.87 | 0.25 | 0.08 |
|  |  |  |  |  |  |  |  |  |  |  |  |  |

**Supplementary Table 2.** *P*-values for high confidence miR-eQTL (FDR < 0.05) identified in this study in GWAS of 11 neurodevelopmental, neurological or psychiatric conditions: Alzheimer’s disease (AD; Bellenguez et al, 2022), anorexia (Watson et al, 2019), anxiety disorders (Otawa et al, 2016), attention deficit hyperactivity disorder (ADHD; Demontis et al, 2019), autism (AUT; Grove et al, 2019), bipolar disorder (BD; Mullins et al, 2021), depression (Dep; Howard et al, 2019), obsessive-compulsive disorder (OCD; IOCDF-GC and OCGAS, 2018), post-traumatic stress disorder (PTSD; Nievergelt et al, 2019), schizophrenia (SZ; Trubetskoy et al, 2022) and Tourette's Syndrome (TS; Yu et al, 2019). The *P*-value in bold surpasses the Bonferroni-corrected threshold for screening 11 traits for 30 miR-eQTL (*P* = 1.5 X 10^-4^).

**References**

Bellenguez, C., Küçükali, F., Jansen, I. E., Kleineidam, L., Moreno-Grau, S., Amin, N., Naj, A. C., Campos-Martin, R., Grenier-Boley, B., Andrade, V., Q., et al (2022) New insights into the genetic etiology of Alzheimer's disease and related dementias. *Nat. Genet.*, **54**, 412–436.

Watson, H. J., Yilmaz, Z., Thornton, L. M., Hübel, C., Coleman, J. R. I., Gaspar, H. A., Bryois, J., Hinney, A., Leppä, V. M., Mattheisen, M., et al (2019) Genome-wide association study identifies eight risk loci and implicates metabo-psychiatric origins for anorexia nervosa. *Nat. Genet*., **51**, 1207–1214.

Otowa, T., Hek, K., Lee, M., Byrne, E. M., Mirza, S. S., Nivard, M. G., Bigdeli, T., Aggen, S. H., Adkins, D., Wolen, A., et al (2016) Meta-analysis of genome-wide association studies of anxiety disorders. *Mol. Psychiatry*, **21**, 1391–1399.

Demontis, D., Walters, R. K., Martin, J., Mattheisen, M., Als, T. D., Agerbo, E., Baldursson, G., Belliveau, R., Bybjerg-Grauholm, J., Bækvad-Hansen, M., et al (2019) Discovery of the first genome-wide significant risk loci for attention deficit/hyperactivity disorder. *Nat. Genet*., **51**, 63–75.

Grove, J., Ripke, S., Als, T. D., Mattheisen, M., Walters, R. K., Won, H., Pallesen, J., Agerbo, E., Andreassen, O. A., Anney, R., et al (2019). Identification of common genetic risk variants for autism spectrum disorder. *Nat. Genet*., **51**, 431–444.

Mullins, N., Forstner, A. J., O'Connell, K. S., Coombes, B., Coleman, J. R. I., Qiao, Z., Als, T. D., Bigdeli, T. B., Børte, S., Bryois, J., et al (2021) Genome-wide association study of more than 40,000 bipolar disorder cases provides new insights into the underlying biology. *Nat. Genet*., **53**, 817–829.

Howard, D. M., Adams, M. J., Clarke, T. K., Hafferty, J. D., Gibson, J., Shirali, M., Coleman, J. R. I., Hagenaars, S. P., Ward, J., Wigmore, E. M., et al (2019). Genome-wide meta-analysis of depression identifies 102 independent variants and highlights the importance of the prefrontal brain regions. *Nat. Neurosci*., **22**, 343–352.

International Obsessive Compulsive Disorder Foundation Genetics Collaborative (IOCDF-GC) and OCD Collaborative Genetics Association Studies (OCGAS) (2018) Revealing the complex genetic architecture of obsessive-compulsive disorder using meta-analysis. *Mol. Psychiatry*, **23**, 1181–1188.

Nievergelt, C. M., Maihofer, A. X., Klengel, T., Atkinson, E. G., Chen, C. Y., Choi, K. W., Coleman, J. R. I., Dalvie, S., Duncan, L. E., Gelernter, J., et al (2019) International meta-analysis of PTSD genome-wide association studies identifies sex- and ancestry-specific genetic risk loci. *Nat. Commun*., **10**, 4558.

Trubetskoy, V., Pardiñas, A. F., Qi, T., Panagiotaropoulou, G., Awasthi, S., Bigdeli, T. B., Bryois, J., Chen, C. Y., Dennison, C. A., Hall, L. S., et al (2022) Mapping genomic loci implicates genes and synaptic biology in schizophrenia. *Nature*, **604**, 502–508.

Yu, D., Sul, J. H., Tsetsos, F., Nawaz, M. S., Huang, A. Y., Zelaya, I., Illmann, C., Osiecki, L., Darrow, S. M., Hirschtritt, M. E., et al (2019) Interrogating the Genetic Determinants of Tourette's Syndrome and Other Tic Disorders Through Genome-Wide Association Studies. *Am. J. Psychiatry*, **176**, 217–227.

| **Trait** | **miRNA** | **Top SNP** | **Chr** | **A1** | **A2** | **Freq** | **Beta_GWAS_** | **SE_GWAS_** | **P_GWAS_** | **Beta_eQTL_** | **SE_eQTL_** | **P_eQTL_** | **Beta_SMR_** | **SE_SMR_** | **P_SMR_** | **P_HEIDI_** | **N SNPs_HEIDI_** |
| --- | --- | --- | --- | --- | --- | --- | --- | --- | --- | --- | --- | --- | --- | --- | --- | --- | --- |
| Bipolar Disorder | miR-1908-5p | rs174561 | 11 | C | T | 0.31 | 0.067 | 0.01 | 1.83E-11 | 1.05 | 0.14 | 9.26E-14 | 0.063 | 0.012 | **5.78E-07** | 8.45E-02 | 20 |
| Sleep duration | miR-1908-5p | rs174561 | 11 | C | T | 0.31 | 0.013 | 0.0024 | 4.10E-08 | 1.05 | 0.14 | 9.26E-14 | 0.012 | 0.0028 | **9.51E-06** | 4.70E-01 | 20 |
| Irritability | miR-1908-5p | rs174561 | 11 | C | T | 0.31 | 0.013 | 0.0025 | 6.20E-08 | 1.05 | 0.14 | 9.26E-14 | 0.013 | 0.0029 | **1.19E-05** | 1.89E-01 | 20 |
| Depressive symptoms | miR-1908-5p | rs174561 | 11 | C | T | 0.31 | 0.0073 | 0.0013 | 6.16E-08 | 1.05 | 0.14 | 9.26E-14 | 0.0069 | 0.0015 | **1.19E-05** | 1.58E-01 | 20 |
| Right cerebellum exterior | miR-1908-5p | rs174561 | 11 | C | T | 0.31 | 0.037 | 0.0076 | 7.95E-07 | 1.05 | 0.14 | 9.26E-14 | 0.035 | 0.0087 | **3.85E-05** | 1.96E-01 | 20 |
| Cognitive performance | miR-1908-5p | rs174561 | 11 | C | T | 0.31 | 0.013 | 0.003 | 7.84E-06 | 1.05 | 0.14 | 9.26E-14 | 0.013 | 0.0034 | 1.29E-04 | 2.15E-01 | 20 |
| Intelligence | miR-1908-5p | rs174561 | 11 | C | T | 0.31 | 0.012 | 0.0029 | 3.49E-05 | 1.05 | 0.14 | 9.26E-14 | 0.011 | 0.0032 | 2.97E-04 | 3.60E-02 | 20 |
| Left cerebellum exterior | miR-1908-5p | rs174561 | 11 | C | T | 0.31 | 0.033 | 0.0077 | 1.34E-05 | 1.05 | 0.14 | 9.26E-14 | 0.031 | 0.0085 | 1.70E-04 | 1.49E-01 | 20 |

**Supplementary Table 3.** SMR and HEIDI results for traits associated with miR-1908-5p eQTL SNP rs174561. *P*_SMR_ values passing the Bonferroni-corrected threshold are indicated in bold. All traits except intelligence are non-significant for the HEIDI test, consistent with pleiotropy / causality rather than linkage.

**References**

Mullins, N., Forstner, A. J., O'Connell, K. S., Coombes, B., Coleman, J. R. I., Qiao, Z., Als, T. D., Bigdeli, T. B., Børte, S., Bryois, J., et al (2021) Genome-wide association study of more than 40,000 bipolar disorder cases provides new insights into the underlying biology. *Nat. Genet*., **53**, 817–829.

Nagel, M., Watanabe, K., Stringer, S., Posthuma, D., van der Sluis, S. (2018) Item-level analyses reveal genetic heterogeneity in neuroticism. *Nat. Commun*., **9**, 905.

Dashti, H. S., Jones, S. E., Wood, A. R., Lane, J. M., van Hees, V. T., Wang, H., Rhodes, J. A., Song, Y., Patel, K., Anderson, S. G., et al (2019) Genome-wide association study identifies genetic loci for self-reported habitual sleep duration supported by accelerometer-derived estimates. *Nat. Commun*., **10**, 1100.

Baselmans, B. M. L., Jansen, R., Ip, H. F., van Dongen, J., Abdellaoui, A., van de Weijer, M. P., Bao, Y., Smart, M., Kumari, M., Willemsen, G., et al (2019) Multivariate genome-wide analyses of the well-being spectrum. *Nat. Genet*., **51**, 445–451.

Zhao, B., Luo, T., Li, T., Li, Y., Zhang, J., Shan, Y., Wang, X., Yang, L., Zhou, F., Zhu, Z.,et al. (2019) Genome-wide association analysis of 19,629 individuals identifies variants influencing regional brain volumes and refines their genetic co-architecture with cognitive and mental health traits. *Nat. Genet*., **51**, 1637–1644.

Lee, J. J., Wedow, R., Okbay, A., Kong, E., Maghzian, O., Zacher, M., Nguyen-Viet, T. A., Bowers, P., Sidorenko, J., Karlsson Linnér, R., et al (2018) Gene discovery and polygenic prediction from a genome-wide association study of educational attainment in 1.1 million individuals. *Nat. Genet*., **50**, 1112–1121.

Savage, J. E., Jansen, P. R., Stringer, S., Watanabe, K., Bryois, J., de Leeuw, C. A., Nagel, M., Awasthi, S., Barr, P. B., Coleman, J. R. I., et al (2018) Genome-wide association meta-analysis in 269,867 individuals identifies new genetic and functional links to intelligence. *Nat. Genet*., **50**, 912–919.

|  | **Biological Process Gene Ontology Term** | **Adjusted *P*-value** |
| --- | --- | --- |
| GO:0022008 | neurogenesis | 2.11E-16 |
| GO:0051254 | positive regulation of RNA metabolic process | 2.50E-15 |
| GO:0045944 | positive regulation of transcription by RNA polymerase II | 5.85E-14 |
| GO:0099536 | synaptic signalling | 7.44E-14 |
| GO:0007267 | cell-cell signalling | 1.27E-13 |
| GO:1902680 | positive regulation of RNA biosynthetic process | 1.88E-13 |
| GO:0048699 | generation of neurons | 2.00E-13 |
| GO:1903508 | positive regulation of nucleic acid-templated transcription | 2.06E-13 |
| GO:0045893 | positive regulation of DNA-templated transcription | 2.06E-13 |
| GO:0099537 | trans-synaptic signalling | 7.21E-13 |
| GO:0007268 | chemical synaptic transmission | 7.34E-13 |
| GO:0098916 | anterograde trans-synaptic signalling | 7.34E-13 |
| GO:0009890 | negative regulation of biosynthetic process | 1.89E-12 |
| GO:0050804 | modulation of chemical synaptic transmission | 2.31E-12 |
| GO:0099177 | regulation of trans-synaptic signalling | 2.66E-12 |
| GO:0010558 | negative regulation of macromolecule biosynthetic process | 3.47E-12 |
| GO:0031327 | negative regulation of cellular biosynthetic process | 4.05E-12 |
| GO:0030182 | neuron differentiation | 5.84E-12 |
| GO:0010557 | positive regulation of macromolecule biosynthetic process | 1.24E-11 |
| GO:0010648 | negative regulation of cell communication | 8.84E-11 |

**Supplementary Table 4.** Gene Ontology analysis of predicted mRNA targets of miR-1908-5p. Predicted targets were retrieved from TargetScan 8.0 (McGeary et al, 2019) and tested for enrichment in Biological Process terms using g:Profiler (Raudvere et al, 2019). The 20 most significant GO terms are shown.

**References**

McGeary, S. E., Lin, K. S., Shi, C. Y., Pham, T. M., Bisaria, N., Kelley, G. M. and Bartel, D. P. (2019) The biochemical basis of microRNA targeting efficacy. *Science*, **366**, eaav1741.

Raudvere, U., Kolberg, L., Kuzmin, I., Arak, T., Adler, P., Peterson, H. and Vilo, J. (2019) g:Profiler: a web server for functional enrichment analysis and conversions of gene lists (2019 update). *Nucleic Acids Res*., **47**, W191–W198

| **Ensembl gene ID** | **Gene** | **SNP** | **statistic** | ***P*-value** | **Beta** | **FDR** |
| --- | --- | --- | --- | --- | --- | --- |
| ENSG00000165060 | *FXN* | rs174561 | -4.141 | 0.000065 | -0.148 | 0.140 |
| ENSG00000121057 | *AKAP1* | rs174561 | 3.399 | 0.000922 | 0.104 | 0.559 |
| ENSG00000134508 | *CABLES1* | rs174561 | 3.387 | 0.000961 | 0.152 | 0.559 |
| ENSG00000167515 | *TRAPPC2L* | rs174561 | 3.351 | 0.001081 | 0.154 | 0.559 |
| ENSG00000115486 | *GGCX* | rs174561 | 3.295 | 0.001299 | 0.083 | 0.559 |
| ENSG00000082153 | *BZW1* | rs174561 | -3.189 | 0.001831 | -0.074 | 0.656 |
| ENSG00000143919 | *CAMKMT* | rs174561 | 3.108 | 0.002361 | 0.098 | 0.725 |
| ENSG00000006118 | *TMEM132A* | rs174561 | 3.043 | 0.002886 | 0.089 | 0.730 |
| ENSG00000182534 | *MXRA7* | rs174561 | -2.949 | 0.003850 | -0.136 | 0.730 |
| ENSG00000173175 | *ADCY5* | rs174561 | 2.942 | 0.003924 | 0.121 | 0.730 |
| ENSG00000174951 | *FUT1* | rs174561 | -2.932 | 0.004047 | -0.141 | 0.730 |
| ENSG00000153443 | *UBALD1* | rs174561 | -2.930 | 0.004075 | -0.192 | 0.730 |
| ENSG00000244165 | *P2RY11* | rs174561 | 2.887 | 0.004629 | 0.093 | 0.766 |
| ENSG00000108591 | *DRG2* | rs174561 | 2.819 | 0.005654 | 0.082 | 0.794 |
| ENSG00000165118 | *C9ORF64* | rs174561 | -2.800 | 0.005965 | -0.086 | 0.794 |
| ENSG00000176658 | *MYO1D* | rs174561 | 2.767 | 0.006567 | 0.107 | 0.794 |
| ENSG00000184992 | *BRI3BP* | rs174561 | -2.740 | 0.007108 | -0.069 | 0.794 |
| ENSG00000188227 | *ZNF793* | rs174561 | 2.722 | 0.007466 | 0.045 | 0.794 |
| ENSG00000143816 | *WNT9A* | rs174561 | -2.720 | 0.007505 | -0.118 | 0.794 |
| ENSG00000166394 | *CYB5R2* | rs174561 | 2.708 | 0.007766 | 0.211 | 0.794 |
| ENSG00000101444 | *AHCY* | rs174561 | 2.705 | 0.007843 | 0.065 | 0.794 |
| ENSG00000131746 | *TNS4* | rs174561 | 2.692 | 0.008128 | 0.096 | 0.794 |
| ENSG00000183496 | *MEX3B* | rs174561 | -2.652 | 0.009106 | -0.118 | 0.803 |
| ENSG00000198740 | *ZNF652* | rs174561 | -2.638 | 0.009459 | -0.068 | 0.803 |
| ENSG00000011105 | *TSPAN9* | rs174561 | 2.613 | 0.010152 | 0.164 | 0.803 |
| ENSG00000162222 | *TTC9C* | rs174561 | -2.589 | 0.010831 | -0.067 | 0.803 |
| ENSG00000185155 | *MIXL1* | rs174561 | -2.567 | 0.011496 | -0.079 | 0.803 |
| ENSG00000127084 | *FGD3* | rs174561 | 2.563 | 0.011625 | 0.177 | 0.803 |
| ENSG00000165566 | *AMER2* | rs174561 | -2.553 | 0.011954 | -0.067 | 0.803 |
| ENSG00000130844 | *ZNF331* | rs174561 | 2.523 | 0.012961 | 0.055 | 0.803 |
| ENSG00000173404 | *INSM1* | rs174561 | -2.523 | 0.012964 | -0.189 | 0.803 |
| ENSG00000167487 | *KLHL26* | rs174561 | 2.514 | 0.013280 | 0.109 | 0.803 |
| ENSG00000092203 | *TOX4* | rs174561 | 2.509 | 0.013472 | 0.072 | 0.803 |
| ENSG00000154370 | *TRIM11* | rs174561 | 2.507 | 0.013533 | 0.070 | 0.803 |
| ENSG00000138600 | *SPPL2A* | rs174561 | 2.505 | 0.013619 | 0.063 | 0.803 |
| ENSG00000103942 | *HOMER2* | rs174561 | -2.490 | 0.014151 | -0.118 | 0.803 |
| ENSG00000160785 | *SLC25A44* | rs174561 | -2.480 | 0.014562 | -0.083 | 0.803 |
| ENSG00000198435 | *NRARP* | rs174561 | -2.470 | 0.014926 | -0.108 | 0.803 |
| ENSG00000125966 | *MMP24* | rs174561 | -2.459 | 0.015399 | -0.069 | 0.803 |
| ENSG00000125637 | *PSD4* | rs174561 | 2.449 | 0.015784 | 0.122 | 0.803 |
| ENSG00000144401 | *METTL21A* | rs174561 | -2.406 | 0.017681 | -0.056 | 0.803 |
| ENSG00000182580 | *EPHB3* | rs174561 | -2.402 | 0.017865 | -0.129 | 0.803 |
| ENSG00000185838 | *GNB1L* | rs174561 | 2.401 | 0.017899 | 0.138 | 0.803 |
| ENSG00000090006 | *LTBP4* | rs174561 | 2.384 | 0.018711 | 0.107 | 0.803 |
| ENSG00000100302 | *RASD2* | rs174561 | 2.376 | 0.019113 | 0.136 | 0.803 |
| ENSG00000185742 | *C11ORF87* | rs174561 | -2.360 | 0.019902 | -0.148 | 0.803 |
| ENSG00000159692 | *CTBP1* | rs174561 | 2.340 | 0.020946 | 0.072 | 0.803 |
| ENSG00000153012 | *LGI2* | rs174561 | -2.337 | 0.021100 | -0.168 | 0.803 |
| ENSG00000165886 | *UBTD1* | rs174561 | -2.336 | 0.021172 | -0.086 | 0.803 |
| ENSG00000084710 | *EFR3B* | rs174561 | -2.331 | 0.021451 | -0.070 | 0.803 |
| ENSG00000101265 | *RASSF2* | rs174561 | -2.330 | 0.021519 | -0.095 | 0.803 |
| ENSG00000204103 | *MAFB* | rs174561 | -2.329 | 0.021561 | -0.176 | 0.803 |
| ENSG00000107957 | *SH3PXD2A* | rs174561 | -2.327 | 0.021693 | -0.077 | 0.803 |
| ENSG00000204120 | *GIGYF2* | rs174561 | 2.318 | 0.022178 | 0.043 | 0.803 |
| ENSG00000125447 | *GGA3* | rs174561 | -2.306 | 0.022867 | -0.065 | 0.803 |
| ENSG00000189306 | *RRP7A* | rs174561 | 2.305 | 0.022889 | 0.099 | 0.803 |
| ENSG00000172578 | *KLHL6* | rs174561 | -2.284 | 0.024138 | -0.055 | 0.803 |
| ENSG00000160710 | *ADAR* | rs174561 | 2.274 | 0.024783 | 0.053 | 0.803 |
| ENSG00000143878 | *RHOB* | rs174561 | -2.263 | 0.025450 | -0.085 | 0.803 |
| ENSG00000249158 | *PCDHA11* | rs174561 | -2.258 | 0.025783 | -0.086 | 0.803 |
| ENSG00000174576 | *NPAS4* | rs174561 | 2.257 | 0.025869 | 0.270 | 0.803 |
| ENSG00000101194 | *SLC17A9* | rs174561 | 2.254 | 0.026045 | 0.169 | 0.803 |
| ENSG00000250120 | *PCDHA10* | rs174561 | -2.249 | 0.026387 | -0.098 | 0.803 |
| ENSG00000087903 | *RFX2* | rs174561 | 2.246 | 0.026582 | 0.117 | 0.803 |
| ENSG00000072818 | *ACAP1* | rs174561 | 2.245 | 0.026639 | 0.131 | 0.803 |
| ENSG00000162065 | *TBC1D24* | rs174561 | -2.238 | 0.027107 | -0.088 | 0.803 |
| ENSG00000172197 | *MBOAT1* | rs174561 | -2.234 | 0.027401 | -0.143 | 0.803 |
| ENSG00000213380 | *COG8* | rs174561 | -2.233 | 0.027467 | -0.046 | 0.803 |
| ENSG00000116903 | *EXOC8* | rs174561 | -2.231 | 0.027544 | -0.038 | 0.803 |
| ENSG00000083814 | *ZNF671* | rs174561 | 2.229 | 0.027735 | 0.066 | 0.803 |
| ENSG00000116473 | *RAP1A* | rs174561 | -2.208 | 0.029186 | -0.054 | 0.803 |
| ENSG00000100129 | *EIF3L* | rs174561 | 2.203 | 0.029548 | 0.091 | 0.803 |
| ENSG00000073754 | *CD5L* | rs174561 | -2.174 | 0.031720 | -0.220 | 0.803 |
| ENSG00000169071 | *ROR2* | rs174561 | -2.171 | 0.031921 | -0.300 | 0.803 |
| ENSG00000049089 | *COL9A2* | rs174561 | 2.170 | 0.032001 | 0.149 | 0.803 |
| ENSG00000170776 | *AKAP13* | rs174561 | 2.166 | 0.032299 | 0.053 | 0.803 |
| ENSG00000102996 | *MMP15* | rs174561 | 2.165 | 0.032407 | 0.089 | 0.803 |
| ENSG00000188060 | *RAB42* | rs174561 | -2.162 | 0.032617 | -0.085 | 0.803 |
| ENSG00000188542 | *DUSP28* | rs174561 | -2.150 | 0.033616 | -0.096 | 0.803 |
| ENSG00000166126 | *AMN* | rs174561 | 2.150 | 0.033627 | 0.139 | 0.803 |
| ENSG00000169410 | *PTPN9* | rs174561 | 2.149 | 0.033699 | 0.054 | 0.803 |
| ENSG00000135472 | *FAIM2* | rs174561 | 2.147 | 0.033827 | 0.098 | 0.803 |
| ENSG00000156869 | *FRRS1* | rs174561 | 2.146 | 0.033901 | 0.094 | 0.803 |
| ENSG00000126583 | *PRKCG* | rs174561 | 2.143 | 0.034204 | 0.169 | 0.803 |
| ENSG00000156453 | *PCDH1* | rs174561 | 2.142 | 0.034229 | 0.081 | 0.803 |
| ENSG00000174165 | *ZDHHC24* | rs174561 | -2.136 | 0.034728 | -0.067 | 0.803 |
| ENSG00000188191 | *PRKAR1B* | rs174561 | -2.129 | 0.035340 | -0.080 | 0.803 |
| ENSG00000177697 | *CD151* | rs174561 | 2.126 | 0.035622 | 0.078 | 0.803 |
| ENSG00000090924 | *PLEKHG2* | rs174561 | 2.123 | 0.035813 | 0.066 | 0.803 |
| ENSG00000175984 | *DENND2C* | rs174561 | -2.120 | 0.036118 | -0.057 | 0.803 |
| ENSG00000116604 | *MEF2D* | rs174561 | 2.119 | 0.036192 | 0.080 | 0.803 |
| ENSG00000161016 | *RPL8* | rs174561 | -2.119 | 0.036217 | -0.085 | 0.803 |
| ENSG00000134369 | *NAV1* | rs174561 | -2.117 | 0.036333 | -0.068 | 0.803 |
| ENSG00000142798 | *HSPG2* | rs174561 | -2.117 | 0.036403 | -0.131 | 0.803 |
| ENSG00000172824 | *CES4A* | rs174561 | 2.103 | 0.037612 | 0.080 | 0.803 |
| ENSG00000063438 | *AHRR* | rs174561 | 2.096 | 0.038175 | 0.138 | 0.803 |
| ENSG00000129911 | *KLF16* | rs174561 | -2.088 | 0.038946 | -0.103 | 0.803 |
| ENSG00000167371 | *PRRT2* | rs174561 | 2.086 | 0.039094 | 0.081 | 0.803 |
| ENSG00000100532 | *CGRRF1* | rs174561 | -2.084 | 0.039285 | -0.085 | 0.803 |
| ENSG00000157782 | *CABP1* | rs174561 | 2.082 | 0.039492 | 0.151 | 0.803 |
| ENSG00000134061 | *CD180* | rs174561 | 2.079 | 0.039830 | 0.067 | 0.803 |
| ENSG00000164938 | *TP53INP1* | rs174561 | -2.072 | 0.040432 | -0.065 | 0.803 |
| ENSG00000170677 | *SOCS6* | rs174561 | -2.069 | 0.040710 | -0.052 | 0.803 |
| ENSG00000198546 | *ZNF511* | rs174561 | -2.066 | 0.041064 | -0.079 | 0.803 |
| ENSG00000171051 | *FPR1* | rs174561 | -2.055 | 0.042123 | -0.094 | 0.803 |
| ENSG00000198626 | *RYR2* | rs174561 | 2.040 | 0.043567 | 0.204 | 0.803 |
| ENSG00000167291 | *TBC1D16* | rs174561 | -2.040 | 0.043577 | -0.070 | 0.803 |
| ENSG00000073849 | *ST6GAL1* | rs174561 | 2.039 | 0.043654 | 0.065 | 0.803 |
| ENSG00000173950 | *XXYLT1* | rs174561 | -2.038 | 0.043826 | -0.046 | 0.803 |
| ENSG00000120549 | *KIAA1217* | rs174561 | 2.034 | 0.044161 | 0.163 | 0.803 |
| ENSG00000095777 | *MYO3A* | rs174561 | 2.033 | 0.044279 | 0.140 | 0.803 |
| ENSG00000090661 | *CERS4* | rs174561 | 2.026 | 0.045066 | 0.057 | 0.803 |
| ENSG00000001617 | *SEMA3F* | rs174561 | 2.023 | 0.045319 | 0.091 | 0.803 |
| ENSG00000164024 | *METAP1* | rs174561 | -2.021 | 0.045534 | -0.051 | 0.803 |
| ENSG00000168237 | *GLYCTK* | rs174561 | 2.020 | 0.045619 | 0.087 | 0.803 |
| ENSG00000081842 | *PCDHA6* | rs174561 | -2.020 | 0.045690 | -0.084 | 0.803 |
| ENSG00000129933 | *MAU2* | rs174561 | 2.018 | 0.045883 | 0.052 | 0.803 |
| ENSG00000140830 | *TXNL4B* | rs174561 | 2.015 | 0.046220 | 0.071 | 0.803 |
| ENSG00000079246 | *XRCC5* | rs174561 | 2.013 | 0.046434 | 0.052 | 0.803 |
| ENSG00000129003 | *VPS13C* | rs174561 | -2.012 | 0.046540 | -0.045 | 0.803 |
| ENSG00000223802 | *CERS1* | rs174561 | -2.007 | 0.047024 | -0.063 | 0.803 |
| ENSG00000151948 | *GLT1D1* | rs174561 | -2.006 | 0.047115 | -0.057 | 0.803 |
| ENSG00000156253 | *RWDD2B* | rs174561 | -2.006 | 0.047135 | -0.076 | 0.803 |
| ENSG00000114933 | *INO80D* | rs174561 | -1.994 | 0.048417 | -0.049 | 0.803 |
| ENSG00000162946 | *DISC1* | rs174561 | 1.993 | 0.048557 | 0.083 | 0.803 |
| ENSG00000140199 | *SLC12A6* | rs174561 | 1.992 | 0.048683 | 0.046 | 0.803 |
| ENSG00000163531 | *NFASC* | rs174561 | 1.982 | 0.049833 | 0.049 | 0.803 |

**Supplementary Table 5.** *Trans-*eQTL analysis of rs174561 in relation to the expression of predicted mRNA targets of miR-1908-5p in the prenatal human brain. Genotype and gene expression data (corrected for known covariates and latent factors) from 120 brain samples from the second trimester of gestation were obtained from O’Brien et al (2018). *Trans*-eQTL analysis was performed by linear regression using Matrix eQTL (Shabalin, 2012), restricted to the 2150 miR-1908-5p targets predicted by TargetScan 8.0 (McGeary et al, 2019) that were found to be expressed in fetal brain (O’Brien et al., 2018). Only eQTL effects that are nominally significant (*P* < 0.05) are shown.

**References**

O'Brien, H. E., Hannon, E., Hill, M. J., Toste, C. C., Robertson, M. J., Morgan, J. E., McLaughlin, G., Lewis, C. M., Schalkwyk, L. C., Hall, L. S., et al (2018) Expression quantitative trait loci in the developing human brain and their enrichment in neuropsychiatric disorders. *Genome Biol.*, **19**, 194.

Shabalin A. A. (2012) Matrix eQTL: ultra fast eQTL analysis via large matrix operations. *Bioinformatics*, **28**, 1353–1358.

McGeary, S. E., Lin, K. S., Shi, C. Y., Pham, T. M., Bisaria, N., Kelley, G. M. and Bartel, D. P. (2019) The biochemical basis of microRNA targeting efficacy. *Science*, **366**, eaav1741.
